# Antimicrobial Peptides in Farm Animals: An Updated Review on Its Diversity, Function, Modes of Action and Therapeutic Prospects

**DOI:** 10.3390/vetsci7040206

**Published:** 2020-12-18

**Authors:** Rohit Kumar, Syed Azmal Ali, Sumit Kumar Singh, Vanya Bhushan, Manya Mathur, Shradha Jamwal, Ashok Kumar Mohanty, Jai Kumar Kaushik, Sudarshan Kumar

**Affiliations:** Cell Biology and Proteomics Lab, Animal Biotechnology Centre, National Dairy Research Institute, Karnal 132001, India; rohitazad247@gmail.com (R.K.); azmal786@gmail.com (S.A.A.); sumitbiotech.singh@gmail.com (S.K.S.); bhushan.vanya22@gmail.com (V.B.); manya.mathur@gmail.com (M.M.); ishuangel17@gmail.com (S.J.); ashokmohanty1@gmail.com (A.K.M.); jaikaushik.icar@gmail.com (J.K.K.)

**Keywords:** anti-microbial peptide, AMP, farm animals, cow, pig, horse

## Abstract

Antimicrobial peptides (AMPs) are the arsenals of the innate host defense system, exhibiting evolutionarily conserved characteristics that are present in practically all forms of life. Recent years have witnessed the emergence of antibiotic-resistant bacteria compounded with a slow discovery rate for new antibiotics that have necessitated scientific efforts to search for alternatives to antibiotics. Research on the identification of AMPs has generated very encouraging evidence that they curb infectious pathologies and are also useful as novel biologics to function as immunotherapeutic agents. Being innate, they exhibit the least cytotoxicity to the host and exerts a wide spectrum of biological activity including low resistance among microbes and increased wound healing actions. Notably, in veterinary science, the constant practice of massive doses of antibiotics with inappropriate withdrawal programs led to a high risk of livestock-associated antimicrobial resistance. Therefore, the world faces tremendous pressure for designing and devising strategies to mitigate the use of antibiotics in animals and keep it safe for posterity. In this review, we illustrate the diversity of farm animal-specific AMPs, and their biochemical foundations, mode of action, and prospective application in clinics. Subsequently, we present the data for their systematic classification under the major and minor groups, antipathogenic action, and allied bioactivities in the host. Finally, we address the limitations of their clinical implementation and envision areas for further advancement.

## 1. Introduction

Globally, intensive livestock farming has led to a rise in the consumption of antibiotics. Imprudent and excessive use of antibiotics in livestock has resulted in an increase in the incidences of antibiotic resistance in several pathogenic bacterial strains and contamination of dairy and meat products with higher levels of antibiotic residues, posing a very serious threat to human health. In 2010, China, the United States, Brazil, India, and Germany were the five top countries in terms of antimicrobial consumption in food animals with 23%, 13%, 9%, 3%, and 3% of total consumption, respectively. By 2030, the expected rise in this figure is projected to be China (30%), the United States (10%), Brazil (8%), India (4%), and Mexico (2%) [1]. The multi-cellular organism has the natural capacity to derive and devise defense strategies against pathogenic attacks. The majority of antimicrobial peptides (AMPs) originate from the cleavage of critical proteins during the process of protein turnover by several proteases. AMPs are evolutionarily conserved weapons against pathogens in almost every organism. These are also known as “host defense peptides” because of their involvement in conferring non-specific innate immunity to the host. Systematic screening of peptides in various species has led to the discovery of many AMPs, some of which are proven to have very effective broad-spectrum activities.

AMPs have been found in almost every stratum of life, ranging from arthropods, amphibians, and reptiles to mammals [2,3,4,5]. It is not surprising that, to date, more than 5000 AMPs have been documented so far, either discovered denovo or synthesized in the lab [6]. Farm animals have constantly been exposed to a heavy dose of antibiotics with improper withdrawal programs. The danger of livestock-associated antimicrobial resistance in humans is on the rise. Although there have been studies on understanding the innate immunity in farm animals, dedicated and systematic studies on AMPs derived from farm animals and their potential as a substitute to antibiotics are not available. In this review, we comprehensively updated the information about AMPs in farm animals, their diversity, chemical characteristics, modes of action, and challenges in their clinical applications.

## 2. Structural Diversity Among AMPs

Nuclear magnetic resonance (NMR) has played a significant role in determining the structural details of AMPs. An analysis of the peptide’s three-dimensional structure has offered a deeper insight into their functions. Two-dimensional NMR methods are mainly used for obtaining three-dimensional structures for small-sized AMPs that can be divided into five classes according to their secondary structure [7]. Although these peptides remain unstructured in the solution, they may adopt specific structural characteristics after coming in contact with the membrane. Four such structural characteristics have been observed, namely alpha-helical, beta stranded, beta-hairpin or loop, and extended conformation. The structures of AMPs, discussed in the later section, are summarised in Table 1. Most of the AMPs discussed here possess either α-helix or β-sheet structure. The structure is either confirmed by NMR, CD, or homology modeling studies, where the atomic structure of the target sequence is constructed from its amino acids and the three-dimensional structure of a template. Most of the AMPs belong to the α-helical conformations. A large number of sequences have been reported in this class, which includes both naturally isolated and chemically synthesized peptides. In an aqueous solution, α-helical AMPs have a linear structure, and upon contact with a bacterial membrane or organic solvent, it forms an amphipathic helical structure [8]. The amphipathic helical structure allows the α-helix AMPs to burrow deep into the phospholipid bilayer and disrupt the membrane integrity. The Myeloid antimicrobial peptide from different farm animals, namely BMAP-27, SMAP-29, and PMAP-23, all possess anα-helix as their secondary structure [9,10,11]. Cecropin P1, eCATH1, eCATH2, and eCATH3, members of the cathelicidin family, also possess α-helical conformation [12,13,14].

In general, β-sheet AMPs are cyclic molecules composed of at least two antiparallel β-sheets stabilized by intramolecular disulfide bonds. The β-sheet peptides are more ordered in an aqueous solution due to their rigid structure and do not undergo a drastic conformation shift like helical peptides upon membrane interaction [15]. Unlike α-helix, the mechanistic model behind the β-sheet antimicrobial activity is not well understood. The β-sheet peptides contain primarily plant defensins, mammal α defensins and β defensins (BNBDs, Bovine β defensin-1, porcine β-defensin), insect defensins, proline-rich antibacterial peptides, protegrin, and tachyplins. Defensins are the well-researched β-sheet peptides that are formed in neutrophils, macrophages, and epithelial cells as inactive precursors [7,16]. Protegrin groups of AMPs from porcine have a well-elucidated structure. Members of the protegrin group, PG-1 PG-2 PG-3, and PG-5are composed of two antiparallel β-sheets connected by a β turn [17,18,19].

Loop AMPs have a loop structure stabilized by amide, disulfide, and isopeptides bonds. Thanatin, a prominent member of this group is isolated from the spinning soldier bug. A single disulfide bond between residue 11 and 18 stabilizes and offers thanatin its characteristic structure [20].

Extended peptides lack the classical secondary structure. Oftentimes, they are rich in certain amino acids, including residues of glycine, arginine, tryptophan, proline, and histidine [21,22]. The structure is stabilized by hydrogen bond and Vander Waal interactions with the phospholipid bilayer. Indolicidin has an extended poly-L-proline II helix as a secondary structure; however, in a neutral DPC environment, the peptide backbone takes a boat shape [23,24]. Several AMPs do not belong to any of these classes, and some appear only in aggregated form or when communicating with the membrane [25]. The plant-derived circulin A is a clear example of this, consisting of a combined α-helix and β-sheet structure that forms the cyclic cysteine knot [21]. Proline-rich Bactenecins such asBac-5 and Bac-7, identical to cathelicidins and arginine-rich PR-39, fall into this category [26].

## 3. Biological Function and Mode of Action

Farm animals harbor a variety of AMPs that help them to counter invading pathogens. Some of these AMPs have been utilized in studies where they were characterized, and considerable experimental evidence, discussed in later sections, suggest that these AMPs have activity against a wide range of pathogens. Some AMPs of animal origin, such as Indolicidin from bovine, have reached clinical II/III phase trials against several conditions. While most of the peptides have been actively tested against human-related conditions in clinical trials, their involvement in veterinary medicine is almost negligible. Most of the studies concerning AMPs against animal diseases and conditions are in preclinical studies (Table 1).

**Figure 1:**
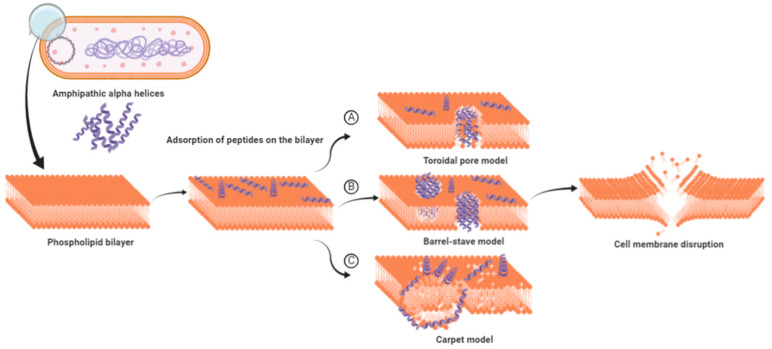
Models proposed for cell membrane disruption by amphipathic α-helix antimicrobial peptides (AMPs). (**A**) The toroidal pore model: hydrophilic region of AMPs are consistently in contact with the polar head groups of the phospholipid bilayer, causing the membrane to curve inward. (**B**) The barrel-stave model: hydrophobic region of AMPs interact with the lipid core of phospholipid bilayer, while the hydrophilic region points inward forming a channel-like structure. (**C**) The carpet model: above critical threshold concentration, AMPs distort the lipid bilayer structure resulting in the micellization of the structure (adapted from references [36,37]).

Depending on the nature of AMPs, the targets can be Gram-positive or G negative bacteria, fungi, or viruses [27,28,29,30,31]. The propensity to bind membranes is a conclusive feature of AMPs, which may or may not be associated with membrane permeabilization [32]. These AMPs employ several mechanisms to wipe out pathogens; most of them rely on the disruption of the cell membrane or alteration in cell membrane permeability, which ultimately relies on various physicochemical parameters possessed by the sequence. While most of the AMPs mainly adopt this approach for killing, others interfere with intracellular functions like DNA and protein synthesis, protein folding, or cell wall synthesis. Physicochemical parameters such as assize, charge, hydrophobicity, and amino acid composition directly affect the ability of the AMPs to insert in the membrane and induce pore formation. The slightest tweak in these parameters might augment the activity of AMPs. These properties facilitate amphipathic α-helix peptides to disrupt bacterial cell membranes by different models, namely, the barrel stave, carpet, and toroidal pore model (Figure 1). In the barrel stave model, peptides interact with one another to form a channel-like structure, while in the toroidal pore model, such peptide-peptide interaction is lacking; toroid pores are rather formed by peptides in a cooperative manner. In the carpet model, instead of forming pores peptides spread across the membrane in parallel orientation disordering the structure, at higher concentrations, these peptides act as a detergent, causing micellization of the phospholipid bilayer.

Cecropin P1 is a prominent example of the carpet model. It preferentially adopts a parallel orientation when reconstituted into a phosphatidylethanolamine/phosphatidylglycerol lipid membrane, as shown by Polarized Attenuated Total Reflectance-Fourier transform infrared(ATR-FTIR) spectroscopy. [12,33]. SMAP-29 from the cathelicidin family also uses the same carpet model as a mode of action as confirmed by fluorescence and molecular dynamics simulations [34]. Collectively, membrane disruption by pore formation is the basic mechanism employed by AMPs to neutralize the bacterial cell. This mechanism is similar to the complement system, which uses membrane attacking complexes to create pores in the cell membrane. Both bacteria and host cells possess phospholipid bilayer cell membrane, so what stops AMPs from attacking the host cell?

**Figure 2:**
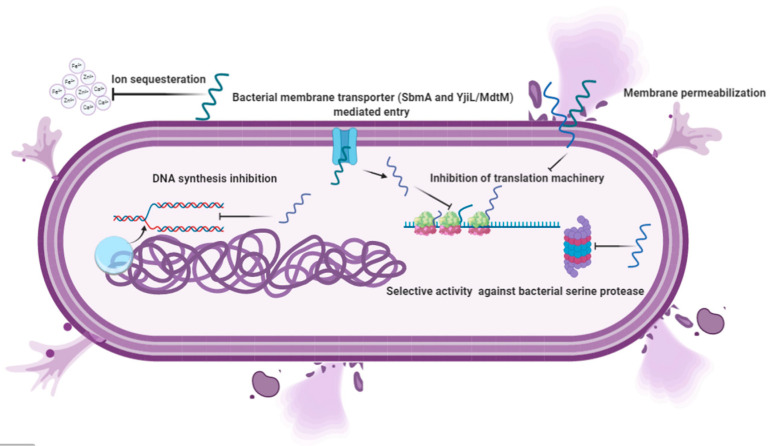
Different mechanisms are used by cathelicidins and β-defensins to eliminate the invading pathogens. After entry, the amphipathic peptides use secondary mechanisms such as inhibition of replication, translation, and sequestration of ions. In addition to membrane lytic action, an attack on these vital processes is life-threatening to bacterial cells and ultimately results in the demise of the cell.

The fundamental basis for antimicrobial action is the difference between bacterial and mammalian cell composition. The outer leaflets of bacterial membranes are populated mainly by negatively charged phospholipids, whereas mammalian cells are mostly composed of neutral zwitterionic phospholipids, such as phosphatidylcholine and sphingomyelin [35]. The Shai-Matsuzaki-Huang model describes the interaction of α-helical AMPs targeting bacterial membranes. It shows the stepwise accumulation of AMPs followed by either integration into the membrane resulting in membrane disruption or diffusion from membrane onto intracellular targets [36,37,38]. These intracellular targets are components of vital processes such as replication and translation and enzymes for sustenance (Figure 2). PR 39, a porcine AMP, exerts its lethal effect by halting the process of translation and DNA synthesis [39]. Similarly, Indolicidin exerts its antimicrobial activity by halting DNA synthesis [40,41]. Marchand and co-workers reported that it inhibits DNA synthesis as well as topoisomerase I, thus employing multiple mechanisms at the DNA level for its antimicrobial activity. It exerts antimicrobial activity in gram-negative bacteria by disrupting the cytoplasmic membrane by forming channels and membrane thinning [23,42]. A similar mechanism for the peptide was reported for a pathogenic fungus *Trichosporon beigelii* [43]. Nevertheless, a different study suggested that even though the peptide caused permeabilization of the bacterial membrane, it was inefficient in lysing bacterial cells [40].

More recently, Indolicidin has been shown to exert its effect by the translocation mechanism, where it induces the release of negatively charged fluorescent dyes such as carboxyfluorescein(CF), calcein, and sulforhodamine B by forming a peptide-dye complex and not by the formation of pores [44]. Some AMPs tend to work by sequestration of ions vital for bacterial survival. Hepcidin and Psoriasin reduce the concentration of iron and zinc ions, respectively, and thus restrict the growth of pathogens [45,46].

**Figure 3:**
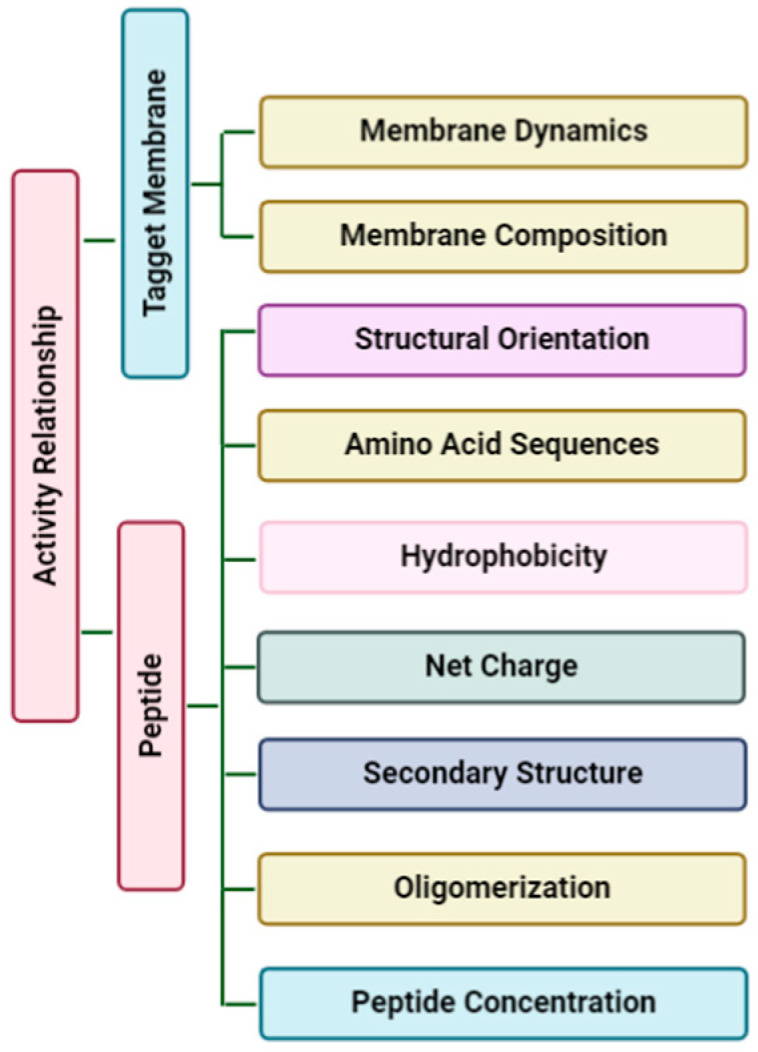
Physicochemical considerations that may impact structure-activity. These factors decide how effectively peptides burrow into the phospholipid bilayer. Membrane dynamics and membrane composition of the target membrane can affect the interaction with the amphipathic peptide.

**Figure 4:**
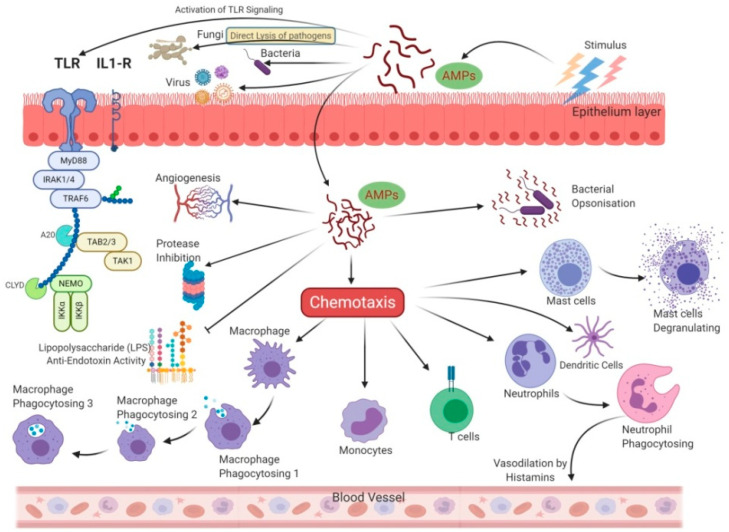
Allied and indirect bioactivities are associated with the AMPs sequence. In addition to direct antimicrobial activity, AMPs also trigger intracellular changes resulting in the activation of anti-inflammatory cytokines, immunomodulation, and promotion of non-specific immunity and wound healing.

It is remarkable that unlike antibiotics, many AMPs are more bactericidal than being bacteriostatic [47]. There are several unique features in the sequence of AMPs, such as net charge, hydrophobicity, secondary structure, etc., which stimulates additional anti-infective support in the host to fight against infection (Figure 3). In addition to having a direct effect on the target organism, AMPs can also act as a potent immunomodulator (Figure 4). They act as effector molecules of the innate immune system and influence diverse processes such as phagocytosis, cytokine release, and cell apoptosis [48,49]. They also act as a chemotactic factor, assisting in recruitment and aggregation of immune cells at the site of inflammation [50,51], promoting angiogenesis [52,53], and inducing wound healing [54] by cytokine release and cell proliferation [55,56].

AMPs in farm animals can be broadly grouped into two leading families, namely cathelicidins and defensins. The cathelicidin family comprises small cationic AMPs that are stored in neutrophils and macrophages. They are part of the innate immune system and are generally proteolytically active proteins. Members of this family have a conserved cathelicidin domain. Defensin is a family of AMPs consisting of six cysteine residues that form three disulfide bonds. It includes three subfamilies, namely α-defensins, β-defensins, and θ-defensins, and these differ from each other in the arrangement of disulfide bonds. These are found in tissues that are involved in host immune response against microbial infections and are abundant in leukocyte granules. The following sections describe the AMPs in these classes in different farm animals comprehensively, which are summarised in Table 1.

## 4. Bovine AMPs

A large number of peptides have been identified in bovines that showed immunity against the broad-spectrum activity of pathogens. The lethal action towards pathogens is mostly due to membrane permeabilization activity, whereas other possesses secondary activities. These secondary activities help the peptides to disrupt and halt the vital functions of the pathogen The bovine AMPs can be broadly classified into two groups, namely, cathelicidins and β-defensins. We have already discussed structural diversity and their mode of action in an earlier section and will explore the in vitro studies carried out against a variety of pathogens.

### 4.1. Bovine Cathelicidins

#### 4.1.1. Indolicidin

Indolicidin belongs to the family of cathelicidin AMPs. It was first discovered in bovine neutrophils as a tridecapeptide and showed a bactericidal effect [57], including its action on *Staphylococcus aureus* [58]. Thus far, seven protein-coding cathelicidin genes have been identified, which are traced to a single cluster on chromosome 22 [59]. Indolicidins are rich in tryptophan residues and cause cytoplasmic disruption in bacteria by forming channels. When compared to its phenylalanine analog, it was found that the presence of a tryptophan residue gives hemolytic activity to the peptide [60]. Treatment of pathogenic *C. albicans* with Indolicidin coated gold nanoparticles (Indolicidin–AuNP) inhibits its ability to produce biofilm by reducing the expression of biofilm-related genes [61]. Another in vitro study reported that Indolicidin–AuNP increases the level of Reactive Oxygen Species (ROS), which results in DNA damage in *S. Cerevisiae* [62].

#### 4.1.2. Bovine MyeloidAntimicrobialPeptides (BMAPs)

Bovine Myeloid AMPs are a series of variable length peptides that belong to the cathelicidin family and show potent antimicrobial activity against a wide range of pathogens. BMAP-18, BMAP-27, and BMAP-28 are the main AMPs belonging to this group. BMAP-27 and BMAP-28 are encoded by CATHL6andCATHL5 genes, respectively. These peptides kill bacteria by forming small channels in the membrane resulting in the release of small ions rather than by membrane disruption [39].

#### BMAP-27

BMAP-27 is bovine cathelicidin; it exhibits broad-spectrum antimicrobial activity against Gram-negative and Gram-positive bacteria, viruses, and fungi, but may also induce some toxicity to erythrocytes [63,64].In addition to its strong LPS (Lipopolysaccharides) neutralizing activity, the peptide also inhibited the formation of biofilm of multidrug-resistant bacterial strains collected from Cystic fibrosis patients [65]. These roles of BMAP-27 were considered useful for the treatment of Gram-negative sepsis [66,67]. Thus, BMAP-27 has a significant role as a potential therapeutic agent against antibiotic-resistant bacteria. The peptide exerts cytotoxicity against human tumor cells by disrupting the membrane, which leads to a Ca2+ influx into the cytosol, ultimately leading to apoptosis [68]. Leucine and phenylalanine zippers are responsible for the cytotoxicity displayed by the peptide, it facilitates the assembly of BMAP 27 on the mammalian cell [69].

#### BMAP-28

BMAP-28 effectively inhibited the growth of pan-drug-resistant *Acinetobacterbaumannii* (PDRAB) by damaging the cell surface with the rapid killing ability [70]. In another study, Lynn and co-workers found that the two stereoisomers of BMAP-28, D-BMAP-28, and the retro-inverso form (RI-BMAP-28), were highly effective against promastigotes and the intracellular amastigotes stages of *Leishmania* life cycles. RI and D isomers of BMAP-28 were unaffected by the proteolytic activity of GP63, a metalloprotease covering the parasite surface. The L-isomer of BMAP-28 was susceptible to degradation by GP63. Takagi et al. (2011) observed the effect of BMAP-28 on methicillin-susceptible *Staphylococcus aureus* (MSSA) and methicillin-resistant *Staphylococcus aureus* (MRSA) [71]. They concluded that for MSSA, the minimal inhibitory concentration (MIC) ranged from 1.25 to 20μg/mL, and for MRSA, the MIC range was 5–20 μg/mL. Similar to BMAP-27, it also exerted cytotoxicity against human tumor cells and healthy proliferating cells [68]. In a cell culture experiment involving RAW 264.7 macrophages, BMAP-28 obstructed the LPS-induced expression of the cytokine gene [72]. The cytotoxic effect of the peptide depends upon its ability to form various transition pores in mitochondria, causing depolarization of the inner mitochondrial membrane [73].

#### BMAP-18

BMAP-18 is derived from its parent peptide BMAP-27. Unlike BMAP-27, it lacks the hydrophobic C-terminal sequence. It showed strong inhibitory activity against several species and life cycle stages of African trypanosomes, fish trypanosomes, and *Leishmania* with reduced cytotoxicity towards mammalian cells as compared to BMAP-27 [74].

A comparative study of BMAP-27 with that of BMAP18 showed that BMAP-18 exerts only antibacterial activity, whereas BMAP-27 showed potent antibacterial as well as anticancer activity. The parent peptide was capable of neutralizing bacteria within 20 min of membrane disruption [9].

#### BMAP-34

BMAP-34, a peptide with α helical structure, is stored as a proform in cytoplasmic granules of neutrophil and exerts broad-spectrum activity against Gram-positive and Gram-negative bacteria [75].

### 4.2. Bovine β-Defensins

#### 4.2.1. Tracheal Antimicrobial Peptide (TAP)

Tracheal AMPs (TAP)is a 38-amino acid peptide that belongs to the β-defensin family and is produced by mucosal epithelial cells of cattle [76]. It was the first defensin to be identified in cattle. One study reported its expression in bovine Mammary Epithelial Cells (bMEC), which was downregulated following *Staphylococcus aureus* infection [77]. Quantitative RT-PCR analysis showed that Pam3CSK4 (a TLR2/1 agonist), IL-17A, and LPS significantly induce the expression of the TAP gene in tracheal epithelial cells [78]. Activation of NF-κB is necessary for the LPS-, Pam3CSK4-, or IL-17A-mediated induction of TAP gene expression [79]. In a study, basal mRNA expression was upregulated in lung tissues from neonatal calves with acute *Mannheimia haemolytica* pneumonia.TAP, NF-κB, and intercellular adhesion molecule 1 expression were also upregulated after infection, among which the expression of TAP and IL-8 were highly correlated [80]. The expression ofTAP and β-defensin in a tightly regulated manner counter bacterial pneumonia.

Bovine origin TAP was expressed in transgenic mice using an expression vector under the control sequences from the murine whey acidic protein (WAP) gene. The bTAP was then later purified by acid precipitation, RP-HPLC, and ion-exchange chromatography with proposed activity against *Escherichia coli* [81]. Studies have shown the in vitro antibacterial activity of the peptide against *Escherichia coli*, *Staphylococcus aureus*, *Klebsiella pneumonia, Pseudomonas aeruginosa*, *Candida albicans*, *Mannheimia haemolytica*, *Histophilus somni*, *Pasteurella multocida*, and *Mycoplasma bovis* [76,81,82].

#### 4.2.2. Lingual Antimicrobial Peptide (LAP)

LAP, a β-defensin, was first isolated from the squamous epithelium of the bovine tongue. It exerts broad-spectrum antimicrobial activities against Gram-positive and Gram-negative micro-organisms, as well as antifungal activity [82]. Immunolocalization studies showed the presence of LAP in the stratum corneum of the stratified squamous epithelium of the tongue, esophagus, rumen reticulum, omasum, chief cells of gastric glands of the abomasum, and mammary alveolar tissue of cattle [83,84]. It is constitutively expressed with other β defensins in mammary lymph nodes [85]. Its expression increased in the mammary gland following coagulase-positive or negative staphylococci infection and vitamin D treatment [86,87].

Studies confirmed a positive correlation between Somatic Cell Count (SCC) in milk and LAP expression, suggesting it as an indicator of SCC [88,89]. Long term high concentrate diet feeding in lactating cows led to an increased translocation of rumen derived LPS into the bloodstream activating enhanced LAP synthesis via the NF-κB signaling pathway [90]. Moreover, challenging mammary glands with LPS increased the LAP level, which exerted a synergistic effect with the lactoperoxidase enzyme [91]. All these studies indicate the decisive role played by LAP in providing innate immunity against invading pathogens in the mammary gland and digestive tract.

#### 4.2.3. BNBD (Bovine Neutrophil β-Defensins)

A variety of β-defensins have been identified in bovine neutrophils. Until now, 13 bovines neutrophil β-defensins have been identified and isolated from neutrophil granules. These AMPs were active against *Staphylococcus aureus* and *Escherichia coli* [92]. BNBD3 or DEFB3 is expressed in various tissues. Treatment of bovine monocyte culture with lipopolysaccharide increased its expression. It enhanced the cell-mediated immune response when co-administered as a fusion with glycoprotein D, a protective agent [93].

Another variant, BNBD4, also known as DEFB4, is constitutively expressed in bovine alveolar tissues in a large amount. It is derived from a prepropeptide that is 63 amino acids long and is converted to a 41 amino acid long mature peptide. Its expression was higher in both the early and late lactation stages of bovine. Its expression increased after infecting mammary glands with coagulase-positive *Staphylococci* as compared to coagulase-negative *Staphylococci* [94]. Another β-defensin known as BNBD5 or DEFB5 was reported, and it resembled BNBD4. It showed increased expression during intramammary infections. The expression of BNBD5 was observed to be high during late lactation stages. Besides their role in countering mammary infections, both BNBD4 and BNBD5 are expressed constitutively in the pulmonary macrophages [95]. BNBD12 and BNBD13 are derived from a common precursor of 60 amino acids, and the mature peptides are composed of 38 and 42 residues, respectively [96].

#### 4.2.4. Enteric β–Defensins

Bovine enteric β defensin (EBD) has been identified in the epithelial cells of bovine small intestine and colon. The EBD prepropeptide and mature peptide show a 72% and 67% similarity to those of TAP, respectively. Tarver et al. (1998) showed that in calves infected by intestinal bacteria *Cryptosporidium parvum*, the EBDs expression was highly inducible, and it increased 5- and 10-fold in the intestine of infected animals. EBD mRNA was more abundant in colon tissues as compared to the small intestine tissues. Northern blot analysis revealed that the expression of other β-defensins in the enteric tissues was low as compared to EBDs [97].

#### 4.2.5. Bovine β-Defensin 1

Aono et al. (2006) identified a novel β-defensin that showed more similarity to human β-defensin than to other bovine β-defensins and named it as bovine β-defensin 1gene (bBD1). It contains two exons and one large intron, which resulted in 69 amino acid propeptides. The translated product exhibits a strong response against *Escherichia coli* but was less effective against *Staphylococcus aureus* infections. It was expressed in the urogenital tract, suggesting that it might have a role in protecting the reproductive tract from a Urinary Tract Infection [98].

### 4.3. Bovine Psoriasin

Psoriasin is an important bovine antimicrobial protein. It is homologous to the human Psoriasin, another name for the calcium-binding S100A7 protein. It was first identified as a respiratory allergen in cattle but played an essential role in providing local defense against bacteria in the udder. It also shows antimicrobial activity, which is observed to be limited against *Escherichia coli*, a mastitis-causing agent. It can be used as an important agent in preventing coliform mastitis [46].

### 4.4. Proline-Rich AMPs: Bac-5 and Bac-7

Two bactenecins, Bac5 and Bac7, were isolated from bovine neutrophils. These sequences are rich in proline and have a molecular mass of 5 and 7 kDa. Both are efficient in suppressing the growth and killing of Gram-negative bacteria, *Escherichia coli*, *Salmonella typhimurium*, and *Klebsiella pneumoniae*. They are also responsible for arresting the growth of *Enterobacter cloacae*. An in vitro assay showed that *Pseudomonas aeruginosa* and *Staphylococcus epidermis* were susceptible to Bac7 but not to Bac5 [99]. Instead of disrupting the bacterial membranes, the proline-rich AMPs (prAMPs) use a non-lytic mode of action. They exert their effects by entering the bacterial cytoplasm via inner membrane transportersSbmA and YjiL/MdtM and inhibit protein synthesis. Bacteria lacking these membrane transporters were resistant to prAMPs [100]. Following their entry into the bacterial cell, peptides bind to the ribosome and prevent the process of translation from proceeding to the elongation phase by blocking the entry site of aminoacyl-tRNA; however, they do not affect DNA and RNA synthesis. Bac5 and Bac7 had little effect on eukaryotic translation as compared to bacterial translation, suggesting that they cause minimal damage to eukaryotic cells [101].

Another study showed that Bac-7 utilized a stereospecificity-dependent mode of action at near MIC value, where the L-enantiomer was more actively internalized into the bacteria. However, it depends on a non-stereospecific lytic mechanism at concentrations higher than MIC value [102].

## 5. Equine AMPs

Unlike others, some AMPs in equine cannot be classified as either cathelicidins or β-defensins, such as hepcidin and Neutrophil Antimicrobial Peptides. These AMPs provide broad-spectrum immunity against a wide range of pathogens. Some of these peptides can be a therapeutic alternative in veterinary medicine such as the treatment of rhodococcosis by eCATH1(discussed in the following section). While many sequences have therapeutic potential, none of them represent the group in the clinical phase of development.

### 5.1. Equine Cathelicidin

Skerlavaj and co-workers reported three putative horse myeloid cathelicidin, namely, eCATH1, eCATH2, and eCATH3, and their cDNA sequences, each peptide having α-helical conformation confirmed by circular dichroism measurement. In vitro studies revealed potent broad-spectrum antimicrobial activity for eCATH1; however, the activity of eCATH2 was somewhat restricted. Both eCATH2 and eCATH3 were produced as propeptides in neutrophils and were cleaved to form mature proteins after neutrophil activation. The activity of eCATH3 showed a strong dependence on salt concentration, as revealed by the efficient killing of bacterial and fungal species in low ionic strength media, which was inhibited in the presence of physiological salt medium [14].

The eCATH1 showed antimicrobial activity against equine isolates of *Rhodococcus equi*, the causal agent of rhodococcosis in foals and showed, *E. coli*, *S. enterica*, *K. pneumoniae*, and *Pseudomonas spp.* with MICs ranging from 0.5–16 µg/mL [103]. eCATH1 showed an in vitro IC50 of 9.5μM against *Trypanosoma brucei*, *Trypanosoma evansi*, and *Trypanosoma equiperdum* [104].

Equine cathelicidin-derived AMPs, EA-CATH1 and EA-CATH2, were isolated from a constructed lung cDNA library of a donkey using nested PCR-based cloning. Chemically synthesized EA-CATH1 had MIC against Gram-positive bacteria in the range of 0.3–2.4 μg/mL. CD measurement studies showed that EA-CATH1 adopts an α-helical conformation in a 50% trifluoroethanol/water solution, but a random coil in an aqueous solution. The sequence showed serum stability and had no hemolytic activity against erythrocytes. Scanning electron microscope studies showed that the treatment of EA-CATH1 caused a rapid disruption of the *Staphylococcus aureus* (ATCC2592) membrane [105].

### 5.2. Equine Neutrophil Antimicrobial Peptides

The eNAP-1 is a 7.2 kDa cysteine-rich endogenous AMP that was purified from the extracts of cytoplasmic granules of equine neutrophils. The peptide was found to be effective against *Streptococcus zooepidemicus*, an equine uterine pathogen. However, it showed less activity against *Escherichia coli* and *Pseudomonas aeruginosa*. The AMP plays an essential role in phagocyte-mediated host defense during equine infections [106].

Another peptide in this series is eNAP-2, a 6.5 KDa endogenous AMP, which was also isolated from the extracts of cytoplasmic granules of equine neutrophils. It showed activity similar to eNAP-1, as it efficiently killed *Streptococcus zooepidemicus*. Studies showed that eNAP-2 exerted bacteriostatic activity against *Klebsiellapneumoniae* by exerting selective activity against microbial serine protease, namely, Subtilisin A and Proteinase K, without any inhibitory effect on mammalian serine protease, ensuring the killing of pathogens without any cytotoxicity to host cells [107,108].

### 5.3. Equine Hepcidin

Hepcidin is a 25 amino acid peptide produced in the liver. In addition to its role in iron homeostasis, it also showed antifungal and antibacterial effects. Hepcidin is synthesized as 84 amino acid prepropeptide with a 24 amino acid N-terminal signal sequence, targeting the peptide in the endoplasmic reticulum. Posttranslational processing of hepcidin is carried out by proprotein convertases, such as furin, PC7/LPC, PACE4, and PC5/6 [109]. During acute inflammation, the increase in levels of iron supports the growth of pathogens, and hepcidin reduces bacterial survival by restricting the availability of iron. In one study, healthy horses were administered with Freund’s complete adjuvant by intramuscular injection at two-time point, namely, 0 h and 12 h. It was observed that 6 h post-infection, hepcidin mRNA increased and remained high until 18 h. The plasma iron concentration decreased significantly between 16 h and 72 h post-infection as compared to control, suggesting the role of hepcidin in the rapid onset of hypoferremia to restrict the availability of iron for the growth of pathogens [45]. Overexpression of hepcidin in transgenic mice resulted in decreased body iron levels and severe microcytic hypochromic anemia, confirming its role in iron sequestration is central to its activity [110].

A research on donkey identified that the high expression of hepcidin in the liver was found to be similar to the reference gene expression. The study also stated that the mature hepcidin sequence from the donkey exhibited 100% sequence homology to the mature hepcidin sequence from the horse [111].

### 5.4. Equine α and β Defensin

Equine β defensin 1 (eBD-1) expression was first reported in hepatic tissue, after a database search for Expressed Sequence Tags ESTs presumed its expression to be in hepatic tissue. Another study showed its expression in respiratory epithelial tissue [112]. The peptide sequence is conserved among human and porcine orthologues, sharing the highest sequence similarity with porcine β defensin [113].

The horse is the only α-defensin expressing species in the Laurasiatheria group [114]. DEFA1 was the first α-defensin to be characterized in the equine and its expression was found in the small intestine. It displayed homology with Panethcell-specific α-defensins from primates. It showed antimicrobial activity against Gram-positive and Gram-negative bacteria and *Candida albicans* by employing membrane permeabilization as the mechanistic approach [114,115].

## 6. Porcine AMPs

Pigs foster plenty of AMPs, which have been extensively studied in terms of their structure, mechanism, and activities against pathogenic bacteria, virus, and yeast. Like others, porcine AMPs also broadly consist of cathelicidins and β-defensins with broad-spectrum activity. These peptides have shown promising results in pre-clinical studies and some of them are in the clinical phase of development. In addition to antimicrobial activities, these peptides possess bioactivities and immunomodulatory activities, which are discussed in the following section.

### 6.1. Porcine Cathelicidins

#### 6.1.1. Protegrins

Protegrins are a group of arginine and cysteine-rich cationic AMPs. They belong to the cathelicidin family and are composed of 16–18 amino acid residues [116]. These peptides were first isolated from porcine leukocytes. There are five known protegrins (PG-1 to PG-5). They exhibit broad-spectrum activity against Gram-negative and Gram-positive bacteria by disrupting the bacterial membranes and releasing their cellular contents. Inactive proforms of protegrins are stored in neutrophils and are secreted during Phorbol Myristate Acetate stimulation; these inactive proforms get converted into mature protegrins via Neutrophil elastase-mediated cleavage [117]. In addition to antimicrobial activity, these mature protegrins also modulate immune activity and cell migration by activating the insulin-like growth factor-1 receptor (IG1R) [118]. The peptide is expressed in the lung and intestinal tissue of porcine. The infection with Porcine Reproductive and Respiratory Syndrome Virus (PRRSV) significantly suppressed its expression in the lungs [119]. It prevents PRRSV virus attachment in Marc-145 cells and suppressed virus RNA and protein synthesis [120].

A study conducted on crossbred piglets showed that weaning reduced the expression of PG-1 significantly [121], but supplementation of 20 mg/Kg of copper in the diet increased the expression of PG-1 and reduced the cases of diarrhea in them [122]. PG-1 was effective in inhibiting Gram-negative, facultative periodontal pathogens. Three strains, each from *Actinobacillus actinomycetemcomitans* and *Capnocytophaga spp.*, were treated with L- and D-enantiomers of PG-1 and L-enantiomers of PG-2, PG-3, and PG-5. Results showed that both the D-enantiomers and the L-enantiomers of PG-1 were equally effective against strains of *Actinobacillus actinomycetemcomitans* and *Capnocytophaga spp.* with its ED99 ranging from 0.5 to 3 μg/mL and 4 to 19 μg/mL, respectively [123]. Sexually Transmitted Disease (STD)-causing pathogens, such as *Neisseria gonorrhoeae* and *Chlamydia trachomatis*, were also susceptible to the peptide. Electron microscope observations showed damages in the cell membrane of pathogens [124,125]. *Candida albicans*, a pathogenic yeast, were also susceptible to PG-1 and other protegrins in series, namely, PG-2, -3, and -5 were equally effective as a candidacidal [18]. PG-1 showed a significant reduction of three log units of viable CFU of MRSA and *Pseudomonas aeruginosa* in less than 15 min. In vivo studies showed that mice that were previously inoculated with *P. aeruginosa* or *S. aureus* showed a better survival rate after receiving an intraperitoneal injection of PG-1 (0.5 mg/kg of body weight) as compared to the mice in the control group exhibiting 93 to 100% mortality [126].

PG-1 works in a concentration-dependent manner. At a concentration below 4 μg/mL, it destabilizes the membrane edge resulting in a finger-like structure. The sieve-like nanoporous structures are formed at the highest concentration, and at a concentration ≥20 μg/mL, it exhibits the maximum degree of membrane disruption [127]. At a physiological NaCl concentration, PG-1 showed enhanced bactericidal activity [128]. PG-1 exhibits selective hemolytic activity against phosphatidylcholine-rich human erythrocytes as compared to phosphatidylethanolamine-rich ruminant erythrocytes, suggesting that the lipid composition is central to the activity of the peptide [129]. A similar observation was made in another study, where PG-1 selectively targeted anionic lipids as compared to the zwitterionic lipids, under similar experimental conditions [130].

The expression of PG-1 in *Pichia pastoris* yielded ~20 mg pure active PG-1 from the supernatant of 500 mL culture broth. The sequence was cloned in pPICZα-A vector fused with 6X His Tag [131]. The introduction of the matrix metalloproteinase cleavage site allowed the efficient release of the peptide at the site of skin inflammation [132].

The complex secondary structure and antimicrobial activity is a major obstruction in its production in the microbial system. Lee et al. created a chloroplast transformation vector containing GFP sequence and Factor Xa cleavage site to release the peptide from the fusion protein. His-tag was introduced to facilitate the purification of the peptide by affinity chromatography. Confocal microscopy showed the localization of peptides in chloroplasts, and the system yielded an adequate amount of purified peptides from transplastomic plants. In mammals, transgenic mice capable of ectopically expressing PG-1 were produced. Those mice expressing PG-1 exhibited enhanced resistance against Actinobacillussuis infection as compared to their wild-type counterparts [133].

#### 6.1.2. PR39

PR39 is a proline and arginine-rich, 39 amino acid residue-long AMP isolated from pig intestine. Linkage and in situ hybridization mapping studies showed PR39 localization on pig chromosome 13 [134]. Immunolocalization showed its presence in the upper and lower respiratory tract tissue of pigs. Its expression was found in type 2 pneumocytes in alveoli [135]. Alterations in the C terminal region of the peptide affected its antimicrobial activity, whereas no significant loss in activity was observed in the case of truncated N-terminal [136,137]. However, the presence of a charged N-terminus is vital for the activity of the peptide in bacterial as well as mammalian systems [137]. PR39 exerts antimicrobial activity, antiapoptotic, antioxidant, chemoattractant, and angiogenic effects. The primary mechanism underlying its inflammation-induced angiogenic effect is the inhibition of ubiquitin–proteasome-mediated degradation of the hypoxia-inducible factor-1α protein [138]. PR39 exhibited an anti-apoptotic effect on macrophages in which apoptosis was induced by nutrient depletion [139]. It prevented tissue injury by inhibiting the deleterious effect of the NADPH oxidase enzyme. Inhibition was achieved via interactions with Src homology three (SH3) domains of cytosolic component followed by blocking the assembly of the enzyme [140].

It also acts as a calcium-dependent chemoattractant for neutrophils [141]. Various in vitro studies suggested the role of PR39 in host defense against pathogens such as *salmonella choleraesuis* and *Mycobacterium tuberculosis* [142,143]. Transgenic mice ubiquitously expressing PR39 showed high resistance to infection from a highly pathogenic swine isolate of *Actinobacillus pleuropneumoniae* (APP) as compared to wildtype littermates [144].

#### 6.1.3. Prophenin 1

Prophenin-1 (PF-1) from the cathelicidin family is a 79 residues long proline-rich AMP. It was first purified and characterized from the porcine leukocytes. PF-1 was more effective against Gram-negative bacteria as compared to Gram-positive bacteria [145]. One study reported that recombinant PF-2 affected the growth and integrity of *Trichomonas vaginalis* [146]. In primary bone marrow cells, expression of the PF-2 gene is regulated by cytokines GM-CSF and IL-3 and transcription factor PU.1 [147].

#### 6.1.4. Cecropin P1

Cecropin P1 is a 31 residue AMP isolated from the porcine small intestine [148]. Additionally, an interaction study between CecP1 and LPS using CD and NMR showed that the peptides adopted an α-helical in a solution containing LPS [149]. It displayed antiviral activityinMarc-145 cells against Porcine Reproductive and Respiratory Syndrome Virus by inhibiting viral attachment, viral particle release, and by elevating the expression of interleukin-6 [150]. A fusion of porcine β-defensin-2 (pBD-2) and CecP1 was expressed in recombinant Bacillus subtilis that was capable of exhibiting antimicrobial activity against several Gram-negative *(E. coli*, *Salmonella typhimurium*, and *Haemophilus parasuis*) and Gram-positive bacteria (*Staphylococcus aureus*) [151]. Cecropin transgene constructs transfected into the Chinook Salmon Embryo cells (CHSE-214) showed antimicrobial activity against *Vibrio anguillarum*, *Aeromonas hydrophila*, and *Pseudomonas fluorescens* following its integration into the genome [152].

#### 6.1.5. Porcine Myeloid Antimicrobial Peptide

PMAP 23 was first characterized from porcine bone marrow by cDNA cloning from RNA encoding 153 residues polypeptide [153]. The 23 residues C terminal sequence was synthesized, capable of exhibiting antibacterial activity against Gram-positive and Gram-negative bacteria with MIC range of 2–16 µM. [11].

The peptide possesses antifungal activity against *Candida albicans*. The treatment of fungi with a FITC-labeled peptide showed its location on the plasma membrane, which tells volume about its mechanistic process [154]. In line with previous work, the peptide was also tested for its antinematodal activity against *C. elegans*, where it exerts its effect by disrupting the cell membrane structure by creating pores [155]. Storici et al. (1994) identified and cloned a cDNA from pig bone marrow RNA encoding 166 residues polypeptide PMAP36, capable of showing its antibacterial activity against various Gram-positive and Gram-negative bacteria, particularly in *E.coli*, where it permeabilizes the bacterial membrane [153]. Similarly, in another study, the (PMAP-36)2 homodimer gene was cloned and expressed in *P. pastoris* GS115; the resultant rPMAP-36 showed in vitro activity against Gram-positive and Gram-negative bacteria. For examining the in vivo activity of the peptide, 10 mg (dissolved in water)/bird/day were given to 120 7-day-old male Arbor acres broiler. Then, cecal and serum samples were taken for the assay. The serum exhibited an elevated level of IgM, and cecal microflora showed an increase in the population of *Bifidobacterium* and a decreased population of *E. coli* [156].

### 6.2. Porcine β Defensin

Porcine β Defensin gene consists of two exons separated by a 1.5 kilobases intron. The entire length of the gene spans 1.9 kilobases and mapping by fluorescence in situ hybridization studies revealed the location of the gene on porcine chromosome 15q14-q15.1 [157]. Recombinant pBD2 was obtained following cloning and expression of pBD2 cDNA in *Pichiapastoris*, yielding 383.7 mg/L of the peptide with a purity of 93.7%. It showed antimicrobial activity against a wide range of pathogenic pig bacteria, namely, *Streptococcus suis*, *Salmonella choleraesuis*, and *Staphylococcus aureus* with MIC values ranging from 32–64 µg/mL [158]. The peptide also showed broad-spectrum activity against porcine intestinal pathogenic bacteria. It was capable of neutralizing the *Salmonella typhimurium*, *Listeria monocytogenes*, and *Erysipelothrix* within 3 h of treatment at 4–8µM concentration, suggesting its role in defending the intestine from pathogenic invasion [159]. Feed additives containing rpBD2 from the culture supernatant resulted in the reduced incidence of post-weaning diarrhea in piglets [160]. Bao et al. (2015) raised anti-Pbd -2 polyclonal antibodies in the New Zealand white rabbit. These antibodies were used in immunohistochemistry studies, which revealed pBD2 distribution in the tongue, liver, kidney, small intestine, and large intestine of pigs [161].

## 7. CaprineAMPs

Exploration studies of AMPs in caprine are still in its early phase, although attempts have been made to characterize and define them. Mostly, these studies were focused on Bactenecin homologous to the bovine Bac5. Proline-rich AMPChBac5 was isolated from elastase treated extracts of goat leucocytes. It is homologous to Bac5 from bovine and showed broad-spectrum activity against diverse pathogens [162].

ChBac3, another proline-rich AMP, was isolated from the leukocytes of *Capra hircus*. It had a 50% sequence similarity to Bac5, with a broad-spectrum antimicrobial activity even at low salt concentrations. The sequence showed high microbial membrane damaging ability as compared to other proline-rich AMPs. However, it does not exhibit any hemolytic activity against human erythrocytes [163]. Two AMPs designated as mini bactenecin, namely, mini-ChBac7.5Nα and mini-ChBac7.5Nβ, were isolated from the neutrophils of the domestic goat. These AMPs possessed antimicrobial activity against Gram-negative bacteria as wells as drug-resistant strains of bacteria (*Klebsiella spp.*, *Pseudomonas aeruginosa*, and *Acinetobacter baumanii*). They also showed activity against some Gram-positive bacteria (*Listeria monocytogeness* and *Micrococcus luteus*) and claimed to have no hemolytic effect on human red blood cells [164].

## 8. Ovine AMPs

The genes coding for ovine defensins and cathelicidins were mapped to chromosome 26 and chromosome 19, respectively. Two exons on chromosome 26 code for two defensins SBD-1 and SBD-2, and four exons on ch19 code for four cathelicidins, namely, OaBac5, OaBac7.5, OaBac6, and OaBac11 [165].

### 8.1. Ovine Cathelicidins

In addition to ChBac3, Shamova and co-workers also isolated OaBac5α from sheep leukocytes via elastase treatment. It was homologous to caprine ChBac3 and bovine Bac5 and showed broad-spectrum antimicrobial activity as well. OaBac5α was considered as a variant of OaBac5. OaBac5β was another variant isolated from sheep leukocytes and was 20–30% more abundant as compared to OaBac5α. OaBac5 and OaBac7.5 are present in truncated forms OaBac5mini and OaBac7.5mini, respectively [162]. These are proline and arginine-rich peptides that were isolated from sheep neutrophils. OaBac5mini exerted a strong response against Gram-negative bacteria but was not effective against Gram-positive bacteria and yeast. On the other hand, OaBac7.5mini was not effective against most of them. Once inside the bacterial cell, they exerted a bactericidal effect by depolarizing the cytoplasmic membrane [166].

SMAP-29, an AMP from the cathelicidin family, was derived from sheep myeloid RNA. It was observed that the N-terminal amphipathic α-helical region and the C-terminal hydrophobic regions were essential for antibacterial and hemolytic activities of SMAP-29 [10]. It also possessed antibacterial activity against antibiotic-resistant clinical isolates such as MRSA and VERF isolates and was also active against *Cryptococcus neoformans* and *Pseudomonas aeruginosa* [167].

### 8.2. Ovine β-Defensins

Ovine β-defensins have two prominent members SBD-1 and SBD-2 located on chromosome-26 [168]. SBD-1 and SBD-2 are small cationic AMPs that are active against Gram-negative and Gram-positive bacteria. SBD-1 expression was found in the trachea, tongue, and gastrointestinal tract, whereas SBD-2 was present in the only ileum and colon [165]. Expression of SBD-1 and SBD-2 along with IL-8 occurred during *M. haemolytica* infections occurred during. However, the expression of SBD-1 was low in M. haemolytica infection as compared to parainfluenza virus type 3 (PI-3)infections. SBD-1 was primarily expressed in respiratory epithelium instead of leukocytes [169].

Zhao et al. (2011) used the Pichiapastoris vector to express 6-His tagged mature SBD-1. The peptide produced had similar activity as mature SBD-1 and had inhibitory effects on *Escherichia coli*, *Staphylococcus aureus*, *Proteus vulgaris*, *Pseudomonas aeruginosa*, and *Shigella flexneri* [170].

## 9. Milk-Derived AMPs

Milk has always been considered a rich source of proteins and other nutrients. The milk protein is composed of 80% casein and 20% whey protein. Several AMPs have been derived from these proteins, and they have the potential to be used in the pharmaceutical industry. Caseinomacropeptide (CMP) is a biologically active peptide that is derived from κ-casein. It is composed of 64 amino acids and is also known as glycomacropeptide [171]. It has various effects, including inhibiting the adhesion of bacteria and viruses and binding to endotoxins. Kappacin is the non-glycosylated and phosphorylated derivative of CMP, which shows a growth inhibitory effect against *Streptococcus mutans* [172]. It exerts its effect by forming pores in the membrane. Another AMP isolated from trypsin-digested κ-casein is κ-casecidin, which exerts its effects by inhibiting the growth of bacteria. The first AMP isolated from N-terminal bovine αs1-casein after chymosin cleavage is Isracidin. It has inhibitory effects on the growth of Gram-positive and Gram-negative bacteria. It is was observed that this peptide protected udders of cattle and sheep from chronic staphylococcal mastitis, and thus, it was suggested that it might have prophylactic and therapeutic effects [173]. Hayes et al. (2006) identified and reported three AMPs namely produced by *lactobacillus acidophilus* DPC6026 fermentation of bovine casein and were referred to asCaseicin A, Caseicin B, and Caseicin C. It was observed that Caseicin A and B show inhibitory activity against pathogenic strains such as. *sakazakii*(ATCC 12868), which causes meningitis in neonates. Thus, these peptides can be used for the production of therapeutic drugs that can protect against this pathogen [174]. A study conducted by Zuchtet al. (1995) isolated and identified an antibacterial peptide- Casocidin1, which was derived fromαs2-casein bovine milk. This peptide was composed of 39 amino acids and inhibited the growth of *Escherichia coli* and *Staphylococcus carnosus* [175].

Several other AMPs have been derived from the whey protein of milk, such as α-lactoglobulin, which is a globular whey protein present in bovine milk. It shows bactericidal activity after digesting it with chymotrypsin and trypsin. The three fragments that were produced were more active against Gram-positive bacteria [176]. On the other hand, digestion with pepsin and trypsin generated fragments that inhibited metabolic activities in *Escherichia coli*. β-Lactoglobulin is the most abundant whey protein. Several AMPs are generated after proteolytic cleavage of β-lactoglobulin. On digesting β-Lb with trypsin, four fragments were generated, and these showed antimicrobial activity towards Gram-positive bacteria only [177]. They also showed that after the modification of a few amino acids, the activity of these fragments could be extended to Gram-negative bacteria as well. Bovine lactoferrin is a 17–41 amino acid glycoprotein that is secreted in body secretions such as saliva, tears, urine, etc. It shows antimicrobial activity against Gram-positive and Gram-negative bacteria and even against viruses and fungi. It is rich in tryptophan and arginine. It acts by disrupting the outer membrane [178]. The activity of lactoferrin was mainly due to the peptide on N1-domain called lactoferricin. Marieke et al. (2004) identified another peptide in this domain and referred to this as lactoferrampin, and interestingly, this peptide showed more significant activity than lactoferrin. It showed a strong response against *Bacillus subtilis*, *Escherichia coli*, and *Pseudomonas aeruginosa*. However, it did not show a strong response against fermenting bacteria such as *Actinomyces naeslundii* [179].

**Table 1:** Major antimicrobial peptides from farm animals.

AMPs	Source	Activity	Structure	Mode of Action
	**CATHELICIDIN**			
Indolicidin	Bovine neutrophils [58]	Gram-positive and Gram-negative bacteria, yeast, and fungi [59,62,63]	Extended helix [24,25]	Membrane thinning, disruption of the membrane by channel formation, inhibition of DNA synthesis, and topoisomerase 1 [24,41,42,43,44,45]
BMAP-18, BMAP-27, BMAP-28,and BMAP-34	Bovine myeloid cells [40]	Gram-positive and Gram-negative bacteria, viruses, fungi, trypanosomes, and tumor cells [40,64,65,66,67,68,71,72,75,76]	α-helix [9,76]	Formation of small channels in the bacterial membrane resulting in the release of small ions, transition pores in mitochondria, and LPS neutralization [9,40,69,73,74],
Bac-5 and Bac-7	Bovine neutrophil [101]	Gram-positive and Gram-negative bacteria [101]	Combination of α-helix and β-sheet [27]	Inhibits protein synthesis by entering via bacterial inner membrane transporter SbmA and YjiL/MdtM [102,103]
eCATH1, eCATH2, and eCATH3	Equine neutrophils [14]	Broad-spectrum [14,106,107]	α-helix [14]	N.A.
Protegrin	Porcine Lung and intestine [121]	Gram-positive and Gram-negative bacteria, PRRSV, and yeast [18,119,121,122,124,125,126,127,128,135]	β-sheet [17,18,19,20]	Pore formation in the bacterial cell membrane and immunomodulation [120,129]
PR-39	Porcine intestine, upper and lower respiratory tract [137]	Gram-negative bacteria and Mycobacterium tuberculosis [144,145,146]	Combination of α-helix and β-sheet [27]	Halts DNA and protein synthesis by exerting proteolytic activity and acts as a calcium-dependent chemoattractant for neutrophil [40,143,144]
Prophenin 1	Porcine leukocytes [147]	More effective against Gram-negative bacteria and less effective against Gram-positive bacteria [147,148]	β-sheet (homology modeling)	N.A.
Cecropin P1	Porcine small intestine [150]	Gram-positive, Gram-negative bacteria and viruses [152,153,154]	α-helix [12,13]	Disruption of lipid bilayer using the carpet model [12,34]
PMAPs	Porcine bone marrow [155]	Gram-positive and Gram-negative bacteria, fungi, and nematodes [155,156,157,158]	α-helix [11]	Permeabilize bacterial membrane [155,156,157]
ChBac3 and ChBac5	Caprine leucocytes [164]	Broad-spectrum [164,166]	N.A.	Membrane disruption [165]
OaBac5, OaBac7, and variants	Ovine leucocytes [164]	Broad-spectrum [164,168]	N.A.	depolarization of the cytoplasmic membranes [168]
SMAP29	Ovine myeloid cells [10]	Gram-positive and Gram-negative bacteria and yeast [169]	α-helix [10]	Permeabilize bacterial membrane [35]
	**DEFENSINS**			
TAP	Bovine mucosal epithelial cells and mammary epithelial cells [77,78]	Broad-spectrum [77,81,82,83]	β-sheet (homology modeling)	N.A.
LAP	Bovine squamous epithelial cells tongue, esophagus, rumen reticulum, omasum, and chief cells of gastric glands [85,86,87]	Broad-spectrum [84]	β-sheet (homology modeling)	N.A.
BNBDs	Bovine neutrophils alveolar tissue and pulmonary macrophages [96,97]	Broad-spectrum [94,96]	β-sheet (homology modeling)	N.A.
EBD	Bovine epithelial cells of intestine and colon [99]	*Cryptosporidium parvum* [99]	N.A.	N.A.
Bovine β-defensin 1	Urogenital tract [100]	Strong response against Gram-negative bacteria as compared to Gram-positive bacteria [100]	β-sheet (homology modeling)	
Equine β defensin 1	Hepatic tissue and respiratory epithelial tissue [114,115]	Broad-spectrum [115]	N.A	N.A
Equine α-defensin DEFA1	Small intestine [116]	Gram-positive andgram-negative bacteriaandfungi [116,117]	β-sheet	membrane permeabilization
Porcine β Defensin	Tongue, liver, kidney, small intestine, and large intestine [163]	Broad-spectrum [160,161,162]	β-sheet (homology modeling)	N.A.
Ovine β-defensin (SBD1 and SBD2)	Trachea, tongue, and gastrointestinal tract [167]	Gram-positive and Gram-negative bacteria, parainfluenza virus, and *M. haemolytica* [167,171,172]	β-sheet (homology modeling)	N.A.
	NEUTROPHIL ANTIMICROBIAL PEPTIDE			
eNAP-1 and eNAP-2	Equine neutrophils [108]	Broad-spectrum [108,109,110]	N.A.	Selective activity against microbial serine proteases [109,110]
	**PSORIASIN**			
Bovine Psoriasin	Udder	Gram-negative bacteria [47]	N.A.	Reduces bacterial survival by zinc sequestration [47]
	**HEPCIDIN**			
Equine hepcidin	Liver [111]	N.A.	N.A.	Induces hypoferrimia by iron sequestration [46,112]

N.A.—not available; Homology modeling-information collected from ModBase: Database of comparative protein structure models.

## 10. Therapeutic Potential of Farm Animal-Derived AMPs

AMPs derived from animals have shown their effectiveness against various conditions and have either cleared or are in advanced stages of clinical trials (Table 2). Besides their performance in the clinical trial, a particular class of AMPs from animals are evaluated against in vitro and in vivo studies and tested against other conditions such as cancer. Various studies confirm the utility of animal origin AMPs. BMAP-28 incorporated with polyurethane PEGU25 showed an inhibitory effect against *Proteus mirabilis*, a common pathogen in catheter-related urinary tract infection [180]. It was also effective in the killing of Methicillin-Resistant *Staphylococcus aureus* with MIC in the range of 5–20 µg/mL [71]. Besides antimicrobial study, the BMAP-28 administration also resulted in a significant reduction in tumor growth in the TT-xenograft mouse model. In vitro study on human thyroid cancer TT cell line showed that the peptide could induce apoptotic effects in the TT cell line, which were confirmed by Annexin V-fluorescein isothiocyanate/propidium iodide and 3-(4,5-dimethylthiazol-2-yl)-2,5-diphenyltetrazolium bromide assay [181].

Thus far, we discussed the peptides that are still in preclinical studies and are being evaluated for their antimicrobial properties, but some of them have also made their way to clinical studies, where they are being assessed for their safety and efficacy. Administration of MBI-226 (omignan) as a 1% topical gel, an indolicidin derivative from bovine, proved effective against catheter-related infections in Phase 3 clinical trial. The study design will be directed toward the assessment of skin irritation, erythema, edema, purulence, moisture, ecchymosis, abnormally warm tissue temperature, and/or site pain/tenderness after changing the dressing on every third day. Cutanea Life Sciences is currently conducting a phase 2 trial to explore the efficacy of 1.75% omignan topical gel in the treatment of Seborrheic dermatitis (NCT03688971).

Use of SGX942 for the treatment of oral mucositis in patients receiving chemoradiation therapy for the treatment of head and neck cancer by intravenous infusion (IV) is in the clinical phase-3 trial (NCT03237325). The drug contains dusquetide as an active ingredient, which is a synthetic 5 residue sequence derived from Indolicidin with broad-spectrum activity against Gram-positive and Gram-negative bacteria and also augments the activity of standard antibiotics. The study has been planned to administer 1.5 mg/mL of the drug as a 4 min IV infusion (twice/week), within three days after initiating radiation therapy, and will be continued for two weeks after the end of radiation therapy.

IB367 Isegnanis another candidate drug for the treatment of oral mucositis in patients receiving radiation therapy for head and neck cancer. It is derived from protegrin-1 and is being tested for its safety and efficacy (NCT00022373). The study is sponsored by the National Cancer Institute and will be conducted on a total of 504 patients (252 for arm I, 168 for arm II, and 84 for arm III). Arm I patient will receive an oral rinse with isegnan HCL solution six times daily, and treatment will be continued for the scheduled duration of radiotherapy. Arm II patients will receive an oral rinse with oral placebo six times daily for the scheduled duration of radiotherapy. Lastly, Arm III patients will receive standard-of-care supportive treatment. Study plans to assess oral cavity pain, ability to swallow, and weight loss twice weekly and on follow up days 28 and 56.

Phase 2 study for POL7080, a protegrin-1 analog, is being conducted by Polyphor Limited, and it aims to assess pharmacokinetics safety and efficacy of the drug in the patients suffering from Ventilator-Associated Pneumonia (VAP) caused by *Pseudomonas aeruginosa* infection (NCT02096328). Findings are not posted yet for the proposed study. Although clinical studies are being carried out on the use of AMPs in various conditions, these drugs are still very far from any kind of commercial and practical applicability.

## 11. Conclusions

Farm animals harbor a wide range of AMPs. Although it is difficult to confine all of them in the set definition of AMP. Most of them belong to cathelicidins and the β-defensin family. There are similarities and overlap in the AMPs in various livestock species, which is not surprising, as they are evolutionarily conserved. Nevertheless, species-specific uniqueness and preference for certain AMPs over others in a particular species encourages us to explore the sea of AMPs and exploit them as an alternative line of treatment in parallel with antibiotics. Despite a large number of such peptides in various livestock species, it is still a matter of waiting for its real application in clinical settings. It is felt in this review that more clinical trials are needed to capitalize on the virtues of AMPs. Another mystery about such AMPs is their mode of action. Apart from the cell membrane disruption, AMPs employ a wide range of secondary mechanisms of action to neutralize the pathogens. Still, for many of these truly effective AMPs, the finer details about the mechanism of action are only poorly understood. The ability of farm animals to thrive under harsher conditions than that of human beings can be seen to be caused by the extraordinary power of AMPs. It is no wonder that livestock-associated AMPs can prove to be of immense application in human diseases. Besides their antimicrobial activity, these molecules can also serve as an immunomodulator, angiogenic factor, vasodilator, chemoattractant, etc. The presence of AMPs in almost every stratum of life and its contribution to innate immunity makes them a suitable candidate for therapeutics against a wide range of pathogens. Some of the peptides of animal origin have shown promising results and have advanced to clinical trials. To mitigate the emergence of antibiotic-resistant pathogens in the future, AMPs can be seen as the best possible alternative.

## Figures and Tables

**Table 2:** AMPs of farm animal origin in clinical trials.

AMPs	Source	Clinical Phase	Indication	Administration	Clinical Identifier
MBI-226Omignan	Indolicidin(Bovine)	Phase III	Prevention of local catheter site infection and colonization in patients with central venous catheters	Topical gel	NCT00231153
CLS001Omignan		Phase III	Severe papulopustular rosacea	Topical gel	NCT02576860
MX942 (SGX942)		Phase III	Immunomodulation during oral mucositis	Intravenous infusion	NCT03237325
Omignan		Phase II	Seborrheic Dermatitis	Topical Gel	NCT03688971
IB-367Isegnan	Protegrin-1 Derivative	Phase III	Oral mucositis in the patient receiving radiation therapy for head and neck cancer	Oral solution	NCT00022373
POL7080	Protegrin-1Analog	Phase II	Non-cystic fibrosis bronchiectasis caused by Pseudomonas aeruginosa infection.	Intravenous infusion	NCT02096328
